# Cognitive inflexibility moderates the relationship between relief-driven drinking motives and alcohol use

**DOI:** 10.1016/j.abrep.2024.100559

**Published:** 2024-06-27

**Authors:** Lara R. Piccoli, Lucy Albertella, Erynn Christensen, Leonardo F. Fontenelle, Chao Suo, Karyn Richardson, Murat Yücel, Rico S.C. Lee

**Affiliations:** a BrainPark, Monash Biomedical Imaging, The Turner Institute for Brain and Mental Health, Monash University, Melbourne, Australia; b Institute of Psychiatry of the Federal University of Rio de Janeiro, Brazil; c D’Or Institute for Research and Education (IDOR), Rio de Janeiro, Brazil; d Department of Psychiatry, School of Clinical Sciences, Monash University, Australia; e QIMR Berghofer Medical Research Institute, Australia; f The Melbourne School of Psychological Sciences, University of Melbourne, Melbourne, Australia

**Keywords:** Alcohol, Relief, Neurocognition, Drinking motives, Executive functions

## Abstract

**Introduction:**

Drinking motives and neurocognition play significant roles in predicting alcohol use. There is limited research examining how relief-driven drinking motives interact with neurocognition in alcohol use, which would help to elucidate the neurocognitive-motivational profiles most susceptible to harmful drinking. This study investigated the interactions between neurocognition (response inhibition and cognitive flexibility) and relief-driven drinking, in predicting problem drinking.

**Methods:**

Participants completed the Alcohol Use Disorders Identification Test – Consumption items (AUDIT-C) to measure drinking behaviour, and online cognitive tasks, including the Value-Modulated Attentional Capture and Reversal Task (VMAC-R) and the Stop Signal Task (SST). The sample (*N* = 368) were individuals who drink alcohol, which included a subsample (*N* = 52) with problematic drinking, as defined by self-identifying as having a primary drinking problem. Drinking motives were assessed using a binary coping question in the overall sample, and the Habit, Reward, and Fear Scale (HRFS) in the subsample. Moderation analyses were conducted to investigate whether cognitive flexibility and response inhibition moderated relationships between relief-driven motives and drinking.

**Results:**

Cognitive flexibility moderated the relationship between relief-driven motives and drinking (overall sample: β = 13.69, *p* = 0.017; subsample: β = 1.45, *p* = 0.013). Greater relief-driven motives were associated with heavier drinking for individuals with low cognitive flexibility. There was no significant interaction between response inhibition and relief-driven motives.

**Conclusions:**

Relief-driven drinking motives interact with cognitive inflexibility to drive heavier drinking. Greater understanding of these neurocognitive-motivational mechanisms may help to develop more targeted and effective interventions for reducing harmful drinking.

## Highlights

• Response inhibition does not interact with relief motives to drive drinking behaviour.
• Cognitive flexibility interacts with relief motives to drive drinking behaviour.
• People who drink for relief and have low cognitive flexibility tend to drink more.

## 1 Introduction

Drinking motives, or reasons for consuming alcohol, play an important role in the transition from recreational to compulsive drinking (Bresin and Mekawi, 2021, Cooper et al., 2016, Kuntsche et al., 2005). Previous research has identified several distinct drinking motives, including reward-, relief-, and more recently, habit-driven motives, involving drinking out of automaticity, without conscious awareness (Adams et al., 2016, Grodin et al., 2019, Piquet-Pessôa et al., 2019). Motivational models of addiction postulate that there is a shift from the positive reinforcement pathway observed in the early, recreational stages of addiction in which people mostly drink to enhance pleasure (i.e., reward-based motives), to the negative reinforcement pathway observed in later stages of addiction, in which drinking is predominantly driven by relief-based motives, such as to reduce negative emotions (Cox and Klinger, 1988, Koob and Moal, 1997, Robinson and Berridge, 1993). Repeated exposure to alcohol and associated environmental cues results in neuroadaptations that sensitise the brain’s reward circuitry, such that these alcohol cues gain incentive salience and trigger more automatic, model-free, and compulsive drinking behaviours (i.e., habit-based motives) which occur independently of anticipated outcomes (Corbit and Janak, 2016, Cox and Klinger, 1988, Koob and Moal, 1997, Robinson and Berridge, 1993). Contemporary measures of drinking motives now encompass habit motives alongside reward and relief motives to capture this progressive shift from affect-driven, goal-directed drinking to habitual alcohol use (Adams et al., 2016, Grodin et al., 2019, Piquet-Pessôa et al., 2019). In this study, we sought to investigate interactions between drinking motives and neurocognition in predicting harmful drinking behaviours.

The utility of understanding drinking motives is underscored by its association with different patterns of alcohol use and alcohol-related problems and outcomes. For example, in alcohol and other substance addictions, individuals with high levels of relief- and habit-based motives tend to endorse more severe/chronic substance use (i.e., greater levels of dependence and alcohol craving), and poorer mental health outcomes (i.e., greater levels of negative affect, anxiety, and depression; Burnette et al., 2021, Corbin et al., 2013, Mann et al., 2018, Piquet-Pessôa et al., 2019, Sofis et al., 2020). Additionally, motives are considered the most proximal factors influencing addictive behaviours, through which more distal factors, such as personality (Kuntsche et al., 2008, Loose et al., 2018) psychopathology (Collins et al., 2021, Kenney et al., 2018, O'Hare and Sherrer, 2011), and neurocognition (Fadardi & Cox, 2008), exert their effects. There has been extensive research examining personality factors and psychopathology, and their associations with drinking motives. Neurocognition, however, has received far less attention regarding its interactions with different motivational profiles of alcohol use.

There is an abundance of research highlighting the contribution of individual differences in executive functioning to the onset of alcohol use problems (Peeters et al., 2015, Verdejo-García et al., 2008). Specifically, poor cognitive flexibility and impaired response inhibition have been found to predict the quantity and frequency of alcohol consumption, heavy or harmful drinking, alcohol-related problems, and relapse (Finn et al., 2009, Goudriaan et al., 2011, Squeglia et al., 2014). Dual process models of addiction expand on motivational models by proposing that affect-driven motives and impaired executive functions predict heavy drinking in tandem, with motives being more influential when top-down cognitive control processes are weaker (Gladwin et al., 2011, Wiers et al., 2007). In other words, an individual with strong relief-based motives, combined with impaired response inhibition and cognitive flexibility, may be even more likely to over-rely on drinking to cope with stress. This interplay suggests that neurocognitive dysfunction interacts with relief-based motives to influence drinking behaviours.

Indeed, recent studies have suggested that the relationship between drinking motives and harmful drinking may be moderated by neurocognitive factors, albeit with inconsistent findings (Liu et al., 2021, Martins et al., 2018). A large study conducted by Martins et al. (2018) on a community sample of 764 adults who had a recent history of alcohol use (i.e., an average of 2–25 drinks per week, and at least one heavy drinking occasion over the past year), found that individual differences in executive functions – response inhibition, set shifting, and working memory updating – did not moderate the effects of affective drinking motives (reward or relief) on alcohol use, heavy drinking, or alcohol-related consequences (Martins et al., 2018). Conversely, our team recently investigated another executive function referred to as reward-related attentional capture (i.e., attentional bias toward reward cues), and found that it interacted with relief-driven motives to drive problematic drinking, in a sample of ninety adults with a similar recent history of alcohol use (Liu, Yücel, et al., 2021). Perhaps the disparity in these findings stems from variations in the nature of the executive function, with certain executive functions potentially exerting a more significant influence on relief-motivated drinking behaviours than others.

While certain facets of cognitive flexibility, namely set-shifting, have not been found to moderate relief-motivated drinking behaviours (Martins et al., 2018), to extend our recent findings, a facet of cognitive flexibility that should be further explored is the persistence of value-modulated attentional capture, which refers to the tendency of individuals to maintain their attention on cues that are associated with high-value rewards, even when such cues are no longer predictive of positive outcomes (Albertella, Watson, et al., 2019). It can be considered a form of cognitive inflexibility, such that individuals high in persistence of value-modulated attentional capture may have difficulty shifting their attention away from alcohol cues that were previously associated with the rewarding effects of alcohol, even when the rewards are no longer available or beneficial to their goals. This inability to shift attention away from previously rewarding cues, paired with high relief-based motives, may predict harmful drinking through persistence of maladaptive coping strategies despite its negative consequences (Cox and Klinger, 1988, Wiers et al., 2007). Our team recently found this effect at the trait-level, using a trait compulsivity self-report questionnaire to reflect cognitive inflexibility, across two independent samples of people who consume alcohol (Albertella et al., 2020, Liu et al., 2022, Liu et al., 2022). Specifically, we found that trait compulsivity moderated the association between distress-driven impulsivity and problematic drinking in university students and a general community sample (Liu et al., 2022, Liu et al., 2022). It is important, however, to determine whether this effect can be observed at the objective cognitive level, via cognitive flexibility tasks, and in more population-representative samples, which to our knowledge, has not been evaluated in alcohol use, thus far.

We aimed to investigate whether response inhibition and cognitive inflexibility moderate the relationship between relief-driven drinking motives and heavier drinking behaviour in a community sample of Australians who drink alcohol, and a subsample of individuals who self-identified as having a problem with their drinking, to determine if the effect was robust. It was hypothesised that both reduced response inhibition and cognitive flexibility would moderate the relationship between relief-based motives and heavier drinking behaviours.

## 2 Materials and methods

### 2.1 Sample

Participants were recruited online from the Australian community using a demographically stratified approach based on age bracket (i.e., 18–24, 25–34, 35–44, 45–54, 55–64 years old) and gender, to increase representation across the general population (Christensen et al., 2024). Inclusion criteria were individuals who have consumed alcohol in the past three months and are aged between 18 and 65 years old. Exclusion criteria were diagnosis of a neurological condition, history of a psychotic disorder, colour blindness, and non-fluency in English. The sample were 368 individuals who drink alcohol, which contained a subsample of people (*N* = 52) who self-reported as having a problem with their drinking through a binary yes/no question and indicated drinking was their primary addictive behaviour of concern. The subsample was included in the study design to assess the consistency and reliability of the analyses across different subgroups of the population of people who drink alcohol (i.e., people who think that they have a problem with their drinking irrespective of whether they meet strict clinical thresholds).

### 2.2 Measures

A binary single item question: ‘do you drink to cope?’ was used to measure whether individuals consume alcohol to cope, which was adapted from the Drinking Motives Questionnaire (Cooper, 1994).

The Habit, Reward and Fear Scale (HRFS; Piquet-Pessôa et al., 2019) is an 18-item transdiagnostic self-report measure that aims to quantify the affective motivations (fear or reward) and habit features of drinking. Items are rated on a Likert scale from one “strongly disagree” to seven “strongly agree”.

The Value-Modulated Attentional Capture and Reversal Task (VMAC-R) used in this study is a modified game version of the reward-only variant of the original VMAC visual search task (Le Pelley et al., 2015), with an additional reversal phase to assess flexibility of attentional capture (Albertella et al., 2019, Albertella et al., 2019). It forms part of the BrainPark Assessment of Cognition (BrainPAC) app, which is a purpose-built assessment tool that has been validated to measure neurocognitive drivers of addiction (Lee et al., 2023). In brief, VMAC-R follows a soccer game format in which participants are soccer players who are required to locate a target visual stimulus (their teammate) while ignoring distractors (opponent players). Participants must pass the ball to their teammate using the left and right arrow keys. On most trials, the distractors are one of two colours: a non-teammate with fluorescent pink or green hair with these different hair colours denoting the magnitude of reward that may be won on that trial. In the reversal phase, these colour-reward contingencies are reversed, and the player must switch flexibly away from responding via the previous contingencies. VMAC-R score was calculated as the reaction time (RT) for trials with the previously-high-reward distractor minus RT for trials with the previously-low-reward distractor during the reversal phase. Greater VMAC-R scores indicate greater persistence of attentional capture by reward cues (i.e., greater cognitive inflexibility).

The Stop Signal Task (SST) measures response inhibition (Eagle and Robbins, 2003a, Eagle and Robbins, 2003b). The gamified version used in this study involves a battlefield game of medieval soldiers fighting a dragon, where participants are required to replenish the arrow supplies of their computerised teammates on the battlefield. During “go” trials, participants must press the left or right arrow key as quickly as they can to move towards one of two teammates when presented with the go “stimulus” (i.e., a teammate raising their hand). During “stop” trials, the enemy dragon breathes a plume of fire down the battlefield after a stop signal delay (SSD); the stop signal indicating that participants should withhold their response. A longer SSD requires greater response inhibition to stop an initiated “go” response. The main outcome variable is the stop signal reaction time (SSRT), which was calculated from the distribution of responses indexing the average time taken to inhibit an initiated motor response on 50 % of trials.

The Perceived Stress Scale 4 (PSS-4; Cohen, 1988) uses a five-point Likert scale to measure the degree to which situations in one’s life over the past month are perceived as stressful. Higher scores indicate higher levels of perceived stress.

The Alcohol Use Disorders Identification Test – Consumption items (AUDIT-C; Bush et al., 1998) is a measure of drinking behaviour derived from the first three items of the AUDIT (Babor et al., 2001); a 10-item self-report questionnaire used to measure frequency and quantity of alcohol consumption, alcohol dependence symptoms, and alcohol-related problems. Higher AUDIT-C scores indicate heavier drinking.

### 2.3 Procedure

This study is cross-sectional and features baseline data collected as part of a larger online longitudinal study that ran from January 2021 to September 2022 and investigated the neurocognitive drivers of addictive and compulsive behaviours (Christensen et al., 2024). Informed consent was obtained from those eligible and willing to participate. The questionnaires were completed via the Qualtrics survey platform (https://www.qualtrics.com), while the cognitive tasks were completed online (Lee et al., 2023). The overall sample completed the binary single item question to determine their coping-driven motives, which was a less reliable, yet more time-efficient way of capturing drinking motives in a large-scale study. Only the smaller subsample of people with problematic drinking also completed a more reliable and comprehensive questionnaire of their relief-driven drinking motives; the HRFS, which allowed us to control for reward- and habit-driven drinking motives in the analyses. All participants were reimbursed with a $50 electronic Prezzee voucher. Study procedures were approved by the Human Research Ethics Committee at Monash University, Australia (Project: 26088).

### 2.4 Statistical analysis

Statistical analyses were conducted using the IBM Statistical Package for the Social Sciences (SPSS) version 29 at α = 0.05. To examine the moderating roles of cognitive inflexibility and response inhibition in the relationships between relief-driven drinking motives and harmful drinking, moderation analyses with bootstrapping (5,000 samples; Neal & Simons, 2007) were conducted using the PROCESS macro (Hayes, 2012) for SPSS version 29. All continuous variables were mean-centred. In moderation models for the overall sample, the binary drinking to cope variable was entered as the independent variable, executive function (either SSRT or VMAC-R score) was entered as the moderator and drinking behaviour (AUDIT-C) was set as the dependent variable. Age, biological sex, perceived stress, and HRFS habit and reward subscale scores were included as covariates given their significant association with neurocognition and drinking, and thus their confounding potential (Albertella, Watson, et al., 2019). VMAC score was also included as a covariate in the cognitive flexibility analyses as this was the phase before the reversal phase in the VMAC-R task, whereby VMAC score was calculated as RT for high-reward trials minus RT for low-reward trials (Albertella, Watson, et al., 2019). Additional analyses were conducted in a subsample of individuals who identified as having a problem with their drinking and endorsed alcohol use as their primary addictive behaviour of concern. These individuals completed the HRFS to assess their drinking motives. Moderation analyses were conducted with the HRFS fear (relief) subscale entered as the independent variable, and the two other HRFS subscales (habit and reward) entered as additional covariates. The significant interaction was explored with a conditional effect analysis and simple slope tests, with the simple slopes tested and plotted at low (+1 standard deviation) and high (−1 standard deviation) levels of cognitive flexibility.

Data quality was ensured through comprehensive screening protocols and post-hoc data cleaning. Specifically, features of the Qualtrics survey platform identified and excluded bots and fraudulent responses. Implausible responses and poor performance likely due to poor effort were identified and removed though attention check questions, neurocognitive task performances below chance levels (Albertella et al., 2019, Lee et al., 2023), and task-specific cleaning procedures. Statistical outliers on VMAC-R and SST that were ≥3 standard deviations from the mean were removed and multiple linear regression assumptions were met prior to conducting the moderation analyses (Field, 2013). We conducted a post-hoc power analysis using G-Power 3.1 (Faul et al., 2007) to determine whether our subsample of 52 had sufficient statistical power. Results revealed that our subsample had 78% and 99% statistical power to detect medium and large effect sizes, respectively.

## 3 Results

### 3.1 Overall sample

The sample consisted of 368 adults (163 males, 201 females, 4 prefer not to say) who drink alcohol. The mean age was 33.14 years (*SD* = 12.61, range 18–65), 71% were Caucasian, and 21% met the criteria for harmful/hazardous levels of alcohol consumption according to AUDIT criteria (i.e., AUDIT total score of 8 or above; Babor et al., 2001), with a median AUDIT score of 4 for the sample. In total, 14.7% of the sample indicated that they drink alcohol to cope. Refer to Table 1 for sample demographics.

**Table 1:** Sample Demographics.

**Variable**	**Overall Sample of Australians who drink alcohol**	**Subsample of individuals with self-identified problematic drinking**
	**(*N* = 368)**	**(*N* = 52)**
Age (SD)	33.14 (12.61)	32.63 (9.72)
Sex (%)		
Female	201 (54.6 %)	25 (48.1 %)
Male	163 (44.3 %)	27 (51.9 %)
Prefer not to say	4 (1.1 %)	
Ethnicity (%)		
Caucasian	261 (70.9 %)	44 (84.6 %)
Asian	68 (18.5)	3 (5.6 %)
Aboriginal or Torres Strait Islander	4 (1.1 %)	
African	4 (1.1 %)	
Hispanic or Latino	4 (1.1 %)	1 (1.9 %)
Middle Eastern	2 (0.5 %)	1 (1.9 %)
South Asian	15 (4.1 %)	2 (3.8 %)
Other	10 (2.7 %)	1 (1.9 %)
Perceived Stress (SD)	7.22 (3.07)	6.79 (3.32)
Coping-Driven Motives (%)		
Drink to cope	54 (14.7 %)	20 (38.5 %)
Do not drink to cope	314 (85.3 %)	32 (61.5 %)
HRFS Habit (SD)		15.31 (8.64)
HRFS Reward (SD)		23.04 (8.18)
HRFS Relief (SD)		14.90 (8.40)
Median AUDIT Total Score	4	7.5

*Note.* This table reflects the means (standard deviations) of continuous variables and the number of people (percentage of the sample) for categorical variables. Median was reported only for AUDIT total score. The HRFS subscale scores are not shown for the overall sample as only the subsample completed this questionnaire to measure their drinking motives. AUDIT = Alcohol Use Disorder Identification Test; HRFS = Habit, Reward and Fear Scale; SD = Standard Deviation.

There was no significant interaction between drinking to cope and SSRT in association with AUDIT-C, (β = −1.93, *p* = 0.436; Table 2), indicating that response inhibition did not moderate the relationship between drinking to cope and heavier drinking. There was, however, a significant interaction effect between drinking to cope and cognitive flexibility, indexed by VMAC-R scores, in association with AUDIT-C (β = 13.69, *p* = 0.017), indicating that cognitive flexibility moderated the relationship between coping motives and heavier alcohol use. That is, in those who drink to cope, lower cognitive flexibility was associated with heavier drinking.

**Table 2:** Moderation Models for Predicting AUDIT-C in Overall Sample.

**Variable**	**β [95 % CI]**	**SE**	***t***	***p***
**SSRT**				
Sex	−0.402 [−0.793, −0.010]	0.199	−2.02	0.044*
Age	0.002 [−0.016, 0.021]	0.009	0.235	0.814
PSS-4	−0.005 [−0.078, 0.067]	0.037	−0.137	0.891
Drink to Cope	2.71 [2.10, 3.32]	0.310	8.72	0.000***
SSRT	−0.408 [−2.46, 1.65]	1.04	−0.390	0.697
Drink to Cope × SSRT	−1.93 [−6.79, 2.93]	2.47	−0.780	0.436
**VMAC-R**				
Sex	−0.379 [−0.770, 0.012]	0.199	−1.91	0.057
Age	0.000 [−0.017, 0.017]	0.009	0.000	0.999
PSS-4	−0.001 [−0.073, 0.071]	0.037	−0.022	0.983
Drink to Cope	2.66 [2.06, 3.26]	0.306	8.71	0.000***
VMAC	0.417 [−0.339, 1.17]	0.384	1.08	0.279
VMAC-R	−1.92 [−5.96, 2.12]	2.05	−0.935	0.351
Drink to Cope × VMAC-R	13.69 [2.43, 24.95]	5.72	2.39	0.017*

*Note. N* = 368*.* Bootstrap sample size = 5,000. *p < 0.05, **p < 0*.*01, ***p < 0.001. CI = confidence interval; PSS-4 = Perceived Stress Scale (four-item) score; SE = standard error; SSRT = stop signal reaction time on the stop signal task; AUDIT-C = Alcohol Use Disorders Identification Test - Consumption items; VMAC = Value-Modulated Attentional Capture Score; VMAC-R = Value-Modulated Attentional Capture and Reversal Score; β = unstandardised beta coefficient.

### 3.2 Subsample

The moderation results were replicated in a subsample, where a structured measure of relief-driven drinking was utilised (i.e., the HRFS), whilst controlling for other drinking motives (habit and reward). This subsample consisted of 52 adults (27 males; *M* age = 32.63 years, *SD* =, 9.72, range 18–57) who indicated drinking as their primary problematic addictive behaviour and predominantly engaged in reward-driven drinking (*M* = 23.04, *SD* = 8.18), compared to habit- (*M* = 15.31, *SD* = 8.64) or relief-driven drinking (*M* = 14.90, *SD* = 8.40). Of the sample, 85% were Caucasian. Half of the sample met the criteria for harmful/hazardous levels of alcohol consumption, with a median AUDIT score of 7.5 for the sample.

There was no significant interaction between relief-driven drinking and SSRT in association with AUDIT-C (β = −0.083, *p* = 0.835; Table 3). There was, however, a significant interaction effect between relief-driven drinking motives and cognitive inflexibility indexed as VMAC-R scores, in association with AUDIT-C (β = 1.45, *p* = 0.013). This indicated that cognitive inflexibility moderated the relationship between relief-driven drinking and heavier drinking, such that relief-driven drinking was more strongly associated with heavier drinking in those with low cognitive flexibility.

**Table 3:** Moderation Models for Predicting AUDIT-C in Subsample.

**Variable**	**β [95 % CI]**	**SE**	***t***	***p***
**SSRT**				
Sex	0.432 [−0.959, 1.82]	0.690	0.626	0.534
Age	0.024 [−0.059, 0.107]	0.041	0.585	0.562
PSS-4	0.082 [−0.127, 0.291]	0.104	0.792	0.433
Habit	0.189 [0.050, 0.327]	0.069	2.75	0.009**
Reward	−0.019 [−0.137, 0.100]	0.059	−0.317	0.753
Relief	0.045 [−0.111, 0.200]	0.077	0.579	0.565
SSRT	−5.51 [−13.89, 2.87]	4.15	−1.33	0.192
Relief × SSRT	−0.083 [−0.876, 0.711]	0.393	−0.210	0.835
**VMAC-R**				
Sex	0.221 [−0.985, 1.43]	0.598	0.370	0.713
Age	0.016 [−0.059, 0.091]	0.037	0.427	0.671
PSS-4	0.084 [−0.097, 0.264]	0.089	0.938	0.354
Habit	0.108 [−0.024, 0.240]	0.066	1.65	0.106
Reward	−0.011 [−0.107, 0.085]	0.048	−0.224	0.824
Relief	0.132 [−0.006, 0.269]	0.068	1.93	0.060
VMAC	8.79 [−3.58, 21.16]	6.13	1.43	0.159
VMAC-R	10.96 [−0.609, 22.54]	5.74	1.91	0.063
Relief × VMAC-R	1.45 [0.320, 2.59]	0.562	2.59	0.013*

*Note. N* = 52*.* Bootstrap sample size = 5,000. *p < 0.05, **p < 0.01, ***p < 0.001. CI = confidence interval; PSS-4 = Perceived Stress Scale (four-item) score; SE = standard error; SSRT = stop signal reaction time on the stop signal task; AUDIT-C = Alcohol Use Disorders Identification Test - Consumption items; VMAC = Value-Modulated Attentional Capture Task score; VMAC-R = Value Modulated Attentional Capture & Reversal Task score; β = unstandardised beta coefficient.

**Fig. 1:**
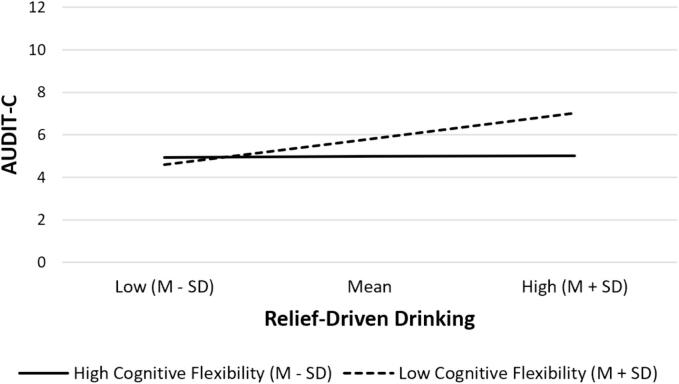
Simple slope plot displaying the moderating role of cognitive inflexibility in the relationship between relief-driven drinking and drinking behaviour. *Note.* M = mean; SD = standard deviation; AUDIT-C = Alcohol Use Disorder Identification Test - Consumption items. The moderating effect is graphed at two levels of cognitive flexibility: low cognitive inflexibility (one SD above the mean) and high cognitive flexibility (one SD below the mean).

The conditional effect analysis revealed that the effect of relief-driven drinking on AUDIT-C was significant at low cognitive flexibility levels (β = 0.210, SE = 0.082, CI = [0.044, 0.376], *p* = 0.014), but not significant at high cognitive flexibility levels (β = 0.053, SE = 0.066, CI [−0.080, 0.187], *p* = 0.425). That is, greater relief-driven drinking was associated with greater AUDIT-C among both low and high cognitively flexibility groups, however, the association was significant only for the low cognitive flexibility group. The effect of relief motives on drinking behaviour at different levels of cognitive flexibility is depicted in (Fig. 1).

## 4 Discussion

As expected, we found that cognitive inflexibility significantly interacted with relief-driven drinking motives to contribute to heavier drinking. Specifically, individuals who drink for relief and have low levels of cognitive flexibility, exhibited greater alcohol use. By contrast, high cognitive flexibility appeared to be a protective factor in people with relief-driven motives. Contrary to our hypothesis, response inhibition did not significantly interact with relief-driven drinking motives in association with heavier alcohol use.

Both relief-driven drinking motives and executive functions, such as cognitive inflexibility and impaired response inhibition are consistently viewed as risk factors for the development and maintenance of addictive behaviours (Christensen et al., 2023, Peeters et al., 2015, Piquet-Pessôa et al., 2019, Verdejo-García et al., 2008). Most studies have investigated associations between these constructs and heavier drinking in isolation. However, increasing evidence supports an interactive effect between relief-based motives and impairments in specific executive functions in contributing to heavy drinking and other problematic behaviours (Liu et al., 2022, Liu et al., 2021). Our finding that reduced response inhibition did not moderate the relationship between relief-driven drinking motives and heavier alcohol use is consistent with previous research (Martins et al., 2018). Perhaps this indicates that whilst response inhibition is a significant cognitive driver of heavier drinking, it may not be directly applicable to relief-driven drinking, where the main drive is to alleviate negative emotions rather than acting on automatic or impulsive urges alone, which instead may be more consistent with habit- and reward-driven motives, respectively. Rather, more relevant may be a facet of impulsivity referred to as negative urgency or distress-driven impulsivity, which comprises of both impulsivity and compulsivity elements (Liu, Rotaru, et al., 2021).

The current study extends our previous findings by showing that cognitive flexibility, in addition to attentional bias (Liu, Yücel, et al., 2021), interacts with relief-driven drinking motives to drive harmful drinking. In other words, people who drink for relief are already at risk of heavy drinking, with attentional bias and cognitive inflexibility further exacerbating their vulnerability to heavier alcohol consumption. This is consistent with our team’s previous research, in which we found that trait compulsivity interacted with distress-driven impulsivity to drive problematic drinking (Liu, Rotaru, Chamberlain, Yücel, et al., 2022). The present study found this effect at the cognitive level, adding further evidence to suggest that individuals with high relief-driven drinking and low cognitive flexibility levels are more susceptible to harmful drinking under negative emotional states, due to impairments in their ability to shift away from maladaptive to healthier coping strategies. Consistently consuming alcohol when stressed can contribute to developing learned associations that drinking relieves such negative emotions, leading to consuming alcohol as a coping mechanism (Robinson and Berridge, 1993). This type of drinking may eventually become maladaptive and lead to alcohol-related problems and other negative consequences. In response to the negative consequences, individuals with high cognitive flexibility may shift their behaviours to alternative, healthier coping strategies, such as exercise or other craving/stress management strategies.

This association has been found in other addictive behaviours (Liu, Rotaru, 2021). Our prior work demonstrated that distress-driven impulsivity significantly interacted with cognitive inflexibility, also measured as persistence of attentional capture by reward cues using the VMAC-R, in association with addiction-like eating (Liu, Rotaru, et al., 2021). Specifically, higher distress-driven impulsivity levels were associated with higher levels of addiction-like eating for individuals with low cognitive flexibility only. Additionally, our other prior work has found a similar trait-level effect in problematic use of the internet (PUI), whereby psychological flexibility moderated the association between distress-driven impulsivity and PUI (Liu, Rotaru, Chamberlain, Ren, et al., 2022). Cumulatively, these findings suggest that this may be a transdiagnostic neurocognitive mechanism observed across a range of addictive behaviours. Future research is required to investigate these cognitive-affective mechanisms in other addictive behaviours, such as compulsive online shopping and problematic pornography use.

A limitation of the present study is that our subsample endorsed higher reward- rather than relief-driven drinking motives, which is associated with more severe drinking behaviours (Piquet-Pessôa et al., 2019). It is important to establish whether the findings can be generalised to more severe alcohol use populations that are farther along in the transition to negatively reinforced drinking. Similarly, our sample was predominantly Caucasian, limiting generalisability to other racial and ethnic groups. While a strength of the study was the inclusion of a subsample to determine if the results could be replicated in individuals with problematic drinking, our subsample was sufficiently powered to detect only large effect sizes. As such, our findings should be interpreted in light of this, as smaller or more modest effects may have gone undetected due to our smaller subsample size. Additionally, the use of a binary single item to assess coping-driven drinking in the overall sample was a limitation due to its limited validity and difficulty in interpretation. Further, the online nature of this study meant that assessment conditions could not be tightly controlled. Despite this, studies have shown that online administration of cognitive tests are a valid alternative to face-to-face tests for assessing neurocognition (McGraw et al., 2000). Lastly, as the current study utilises a cross-sectional design, it does not allow for causal inferences. Longitudinal study designs would allow us to better understand the temporal relationship between relief-driven drinking motives, cognitive inflexibility, and alcohol use outcomes, and the associated pathways to harmful drinking.

This study has notable strengths and clinical implications. Firstly, different cognitive-motivational profiles can statistically predict different patterns of alcohol use and alcohol-related problems. Further elucidating these profiles will help us to understand those most at-risk of harmful drinking and associated problems. Secondly, understanding the cognitive-motivational profiles may help to inform more personalised interventions that can reduce problematic drinking behaviours by targeting specific cognitive-affective mechanisms. For example, administering cue exposure therapy under stressful conditions may reduce persistence of attentional capture by reward cues via extinction of learned associations between the environmental alcohol cues, drinking behaviours, and stress, thereby potentially reducing harmful alcohol use (Kiyak et al., 2023). Teaching alternative coping strategies, such as distress tolerance skills, in response to negative emotional states may also improve drinking outcomes by enabling these skills to be easily activated during future distress, even when cognitive control is impaired (Stasiewicz et al., 2013).

## Funding

The present study was supported by a 10.13039/501100000925National Health and Medical Research Council (NHMRC) Project Grant [APP1162031]. This funding source had no involvement in the study design, interpretation of data, or the writing and submission of the manuscript.

## CRediT authorship contribution statement

**Lara R. Piccoli:** Conceptualization, Formal analysis, Visualisation, Writing – review & editing. **Lucy Albertella:** Writing – review & editing, Supervision, Formal analysis, Conceptualization. **Erynn Christensen:** Validation, Methodology, Investigation. **Leonardo F. Fontenelle:** Writing – review & editing, Validation. **Chao Suo:** Software, Methodology. **Karyn Richardson:** Writing – review & editing. **Murat Yücel:** Writing – review & editing, Validation, Funding acquisition. **Rico S.C. Lee:** Writing – review & editing, Validation, Supervision, Methodology, Investigation, Funding acquisition.

## Declaration of competing interest

The authors declare that they have no known competing financial interests or personal relationships that could have appeared to influence the work reported in this paper.

Lara Piccoli is supported by an Australian Government Research Training Program PhD Scholarship.

Rico Lee has received funding from the NHMRC Investigator Grant funded by the Medical Research Future Fund [APP1193946].

Murat Yücel has received funding from government funding bodies such as the NHMRC, Australian Research Council (ARC), Australian Defence Science and Technology (DST), the Department of Industry, Innovation, and Science (DIIS), the National Institutes of Health (NIH, USA); philanthropic donations from the David Winston Turner Endowment Fund, Wilson Foundation; sponsored investigator-initiated trials including Incannex Healthcare Ltd. Murat also sits on the Advisory Boards of: Centre of The Urban Mental Health, University of Amsterdam; and Enosis Therapeutics. The above funding sources have had no role in the present study design, collection, analysis or interpretation of the data, writing the manuscript, or the decision to submit this paper for publication.

## Data Availability

Data will be made available on request.
